# Circ-ABCB10 knockdown inhibits the malignant progression of cervical cancer through microRNA-128-3p/ZEB1 axis

**DOI:** 10.1186/s12575-021-00154-8

**Published:** 2021-09-07

**Authors:** Wei Feng, Dongya Zhang, Ruitao Zhang

**Affiliations:** Department of Gynecology, The First Affiliated Hospital of Zhengzhou University, NO.1 East Jianshe Road, Zhengzhou, 450052 Henan China

**Keywords:** Circ-ABCB10, miR-128-3p, ZEB1, Cervical cancer, Proliferation, Apoptosis, Invasion

## Abstract

**Aims:**

We focused on the detailed functions of circ-ABCB10 in cervical cancer (CC) development and its mechanisms.

**Background:**

The increasing findings have proposed the central roles of circular RNAs (circRNAs) in the tumorigenesis of various human cancers. Circ-ABCB10 displays promising oncogenic effect in several tumors.

**Methods:**

Circ-ABCB10 and miR-128-3p production levels in CC tissues and cells were tested through RT-qPCR. The association of circ-ABCB10 expression with clinicopathologic parameters of CC patients was statistically analyzed. Cell proliferation, invasion, apoptosis, and epithelial-mesenchymal transition (EMT) were evaluated by MTT, transwell invasion assays, flow cytometry analyses, and western blot examination of EMT markers. The binding activity between miR-128-3p and circ-ABCB10 or zinc finger E-box binding homeobox 1 (ZEB1) was explored through pull-down assay or luciferase reporter assay. The influence of circ-ABCB10 on CC tumorigenesis was evaluated by in vivo xenograft experiments.

**Results:**

The elevated circ-ABCB10 expression was determined in CC tissues and cells. Moreover, higher production level of circ-ABCB10 was close related to lymph-node metastasis, Federation of Gynecology and Obstetrics (FIGO) stage, and tumor size in CC patients. Loss of circ-ABCB10 weakened cell proliferative and invasive abilities, inhibited EMT, and induced apoptosis in CC. Loss of circ-ABCB10 inhibited ZEB1 expression by serving as a sponge of miR-128-3p in CC cells. Circ-ABCB10 sponged miR-128-3p to enhance cell proliferation, invasion, EMT and inhibit apoptosis in CC cells. Xenograft tumor assays confirmed that circ-ABCB10 knockdown inhibited CC tumor growth.

**Conclusion:**

Our study suggests that circ-ABCB10 depletion inhibits proliferation, invasion and EMT and promotes apoptosis of cervical cancer cells through miR-128-3p/ZEB1 axis and represses CC tumor growth.

## Background

Cervical cancer (CC), a huge threat to female health and life, accounts for more than 3% of all new cancer diagnoses and the total cancer deaths worldwide [1]. The morbidity and mortality of CC patients rank the fourth in all malignancies [1]. It was estimated that over 569,000 new CC cases and 311,000 CC-related deaths occurred in 2018 globally [1]. Moreover, the incidence rate and mortality of CC are higher in low-income and middle-income countries (LMIC) relative to developed countries due to the limitations in the management of CC in LMIC [2, 3]. Additionally, patients with recurrent or late-stage CC have a poor prognosis with the overall survival time of less than 2 years [3, 4]. To better control CC, it is imperative to deeply investigate the vital molecular events implicated in CC tumorigenesis and progression [5].

Circular RNAs (circRNAs), produced through back splicing, are a category of covalently closed single-strand RNAs with no or little protein-coding capacity [6, 7]. To date, copious circRNAs have been identified through bioinformatics algorithms and high-throughput sequencing technology in multiple organisms including human [6, 7]. According to growing evidence, moreover, the abnormal expression of circRNAs is implicated in the pathogenesis of many disorders including cancers [8, 9]. Additionally, some circRNAs function as the regulators of CC phenotypes [10]. For instance, the depletion of circRNA_101996 impairs the proliferative, migratory and invasive potential of CC cells through upregulating targeting protein for xenopus kinesin-like protein 2 via sponging microRNA-8075 [11]. Circ_POLA2 knockdown weakens the proliferative, invasive and migratory capacities of CC cells and hinders the growth and metastasis of CC xenograft tumors through microRNA-326/G protein subunit beta 1 axis [12].

The existing body of research on circ-ABCB10 suggests that it is an oncogenic factor in multiple malignancies such as clear cell renal cell cancer [13], epithelial ovarian cancer [14], and oral squamous cell cancer [15]. For instance, circ-ABCB10 knockdown impairs cell proliferative ability, hinders cell cycle progression and facilitates cell apoptosis in breast cancer [16]. Circ-ABCB10 loss suppresses cell proliferation/migration and hampers xenograft tumor growth in non-small cell lung cancer [17]. Nevertheless, the functions and molecular basis of circ-ABCB10 in CC progression are unknown till now.

We became interested in circ-ABCB10 functions after reading these above findings and the present research further explored the influence of loss of circ-ABCB10 on CC tumorigenesis and progression along with its downstream regulatory mechanisms.

## Materials and methods

### Clinical samples

Tumor tissues from CC patients and adjacent normal tissues (> 2 cm from the margin of the tumor) (*n* = 34) were collected and recruited from patients at the First Affiliated Hospital of Zhengzhou University during May 2018 to December 2019 and our protocol was approved by Research Ethics Committee of our hospital and conducted following the Declaration of Helsinki. All pathological specimens were diagnosed by two pathologists. The clinicopathologic parameters of these patients were provided in Table 1.

**Table 1:** Correlation analyses of cric-ABCB10 expression and clinicopathologic parameters of CC patients

Clinical characteristics			Circ-ABCB10			
		*N*	High expression N (%)	Low expression N (%)	Χ^2^	*P*
Age	> 45	7	4 (57.1%)	3 (42.9%)	0.18	0.671
	≤ 45	27	13 (48.1%)	14 (51.9%)		
Histologic type	Squamous cell carcinoma	22	10 (45.5%)	12 (54.5%)	0.515	0.473
	Adenocarcinoma	12	7 (58.3%)	5 (41.7%)		
LNM	Yes	18	12 (66.7%)	6 (33.3%)	4.25	0.039*
	No	16	5 (31.3%)	11 (68.8%)		
FIGO stage	I	15	3 (20.0%)	12 (80.0%)	9.663	0.002*
	II	19	14 (73.7%)	5 (26.3%)		
Tumor size (cm)	> 4	13	10 (76.9%)	3 (23.1%)	6.103	0.013*
	≤ 4	21	7 (33.3%)	14 (66.7%)		

*LNM* lymph node metastasis, *CC* cervical cancer. *P < 0.05

### Cell culture

Siha, CaSki, and Hela cells were ordered from Cell Bank of Chinese Academy of Sciences (Shanghai, China). C33A, SW756 and normal human cervical epithelial cell (HUCEC) lines were obtained from American Type Culture Collection (ATCC; Manassas, VA, USA). CaSki cells were maintained in RPMI-1640 medium containing 10% fetal bovine serum (FBS). C33A, Siha and Hela cells were grown in Minimum Essential Medium containing 10% FBS. SW756 cells were maintained in Leibovitz’s L-15 medium containing 10% FBS in a 100% air incubator at 37 °C. Normal primary cervical epithelial cells were maintained in cervical epithelial cell basal medium (ATCC) added with the contents of cervical epithelial cell growth kit (ATCC). All cells except for SW756 cells were maintained in the incubator containing 5% CO_2_ at 37 °C. FBS and all CC cell medium were ordered from Thermo Scientific lnc. (Waltham, MA, USA).

### Plasmids and cell infection

Lentiviral vector plasmids were purchased from Ke Lei Biological Technology Co., Ltd (Shanghai, China). The lentiviral plasmids overexpressing circ-ABCB10 (pcDNA-circ-ABCB10), miR-128-3p and plasmid interfering circ-ABCB10 were constructed. These plasmids were co-transfected with auxiliary plasmids into 293 T cells to gain viruses expressing these plasmids referring to the manufacturer’s instructions. Finally, the viruses (MOI = 10) were used to infect Hela and C33A cells.

### RT-qPCR assay

Total RNA was isolated from CC tumor tissues and cells by TRIzol reagent (Thermo Scientific). Next, the synthesis of first strand cDNA was conducted using M-MLV Reverse Transcriptase (Thermo Scientific). Subsequently, the quantitative PCR analysis of cDNA was conducted using the SYBR Green Master Mix (Thermo Scientific) on Applied Biosystems 7500 Real-Time PCR System (Thermo Scientific). Circ-ABCB10 or miR-128-3p levels were normalized by GAPDH or U6 snRNA, respectively. Expression patterns of circ-ABCB10 and miR-128-3p were analyzed using the 2^−ΔΔCt^ method.

### MTT assay

The infected cells were seeded into 96-well plates. At 0, 24, 48, or 72 h, 10 µl of MTT solution (5 mg/ml, Beyotime Biotechnology, Shanghai, China) was added into per well. Four hours later, cells were co-incubated with DMSO (150 μl per well). Finally, optical density (OD) values were detected at the wavelength of 490 nm using a microplate reader (Molecular Devices, Sunnyvale, CA, USA).

### Transwell invasion assay

Cell invasive potential was assessed through a transwell chamber (24-well, 8-µm pore size of filter membranes) pre-coated with matrigel (Corning, Corning, NY, USA). Briefly, the upper or low chamber was added with cells (2 × 10^5^) suspended in serum-free medium or medium containing 20% FBS, respectively. Twenty-four hours later, the invaded cells were fixed using the methanol and stained with 0.1% crystal violet (Sigma-Aldrich, St. Louis, MO, USA).

### Western blotting assay

The collected cells and tissues samples were prepared using RIPA lysis buffer (Beyotime Biotechnology) added with protease inhibitor (Thermo Scientific). The total content of protein in the lysates was determined using Pierce BCA Protein Assay Kit (Thermo Scientific). Protein (20 µg/lane) was separated through 10% SDS-PAGE and electro-transferred onto polyvinylidene fluoride membranes (Millipore, Bedford, MA, USA). After blocked with 5% skimmed milk, the membranes were incubated overnight at 4 °C with primary antibody against zinc finger E-box binding homeobox 1 (ZEB1) (1/500 dilution, ab203829), E-cadherin (1/1000 dilution, #3195), N-cadherin (1/1000 dilution, #4061), Vimentin (1/1000 dilution, #5741), snail (1/1000 dilution, #3895) and β-actin (1/2000 dilution, ab8227). After washed, the membranes were probed with HRP-labeled secondary antibody (1/5000 dilution, ab672 or ab6789) for 1 h at 25 °C. ZEB1/β-actin antibody and secondary antibodies were ordered from Abcam lnc. (Cambridge, UK). Other antibodies against EMT markers were obtained from Cell Signaling Technology lnc. (Danvers, MA, USA). Protein bands were developed using Pierce ECL Western Blotting Substrate (Thermo Scientific).

### Cell apoptosis assay

The apoptotic capacity of cells was examined using the Annexin V-PE/7-AAD Apoptotic Detection kit (Sigma). Briefly, cells re-suspended in binding solution were co-incubated with Annexin V-PE and 7-ADD solutions for 10–30 min at 25 °C under the dark conditions. Subsequently, cell apoptotic pattern was analyzed through flow cytometry (BD Biosciences, San Jose, CA, USA).

### Luciferase reporter assay

The corresponding fragment of circ-ABCB10 or ZEB1 3’UTR covering predicted miR-128-3p complementary sites were constructed into pmirGLO vector plasmids by GenePharma Co., Ltd. and generated recombinant plasmids was named as circ-ABCB10-WT or ZEB1-WT reporter. Also, circ-ABCB10-MUT and ZEB1-MUT reporters containing mutant miR-128-3p complementary sequence were also constructed. Next, luciferase reporter was introduced into Hela and C33A cells transfected with miR-Ctrl or miR-128-3p mimics. Forty-eight hours later, dual luciferase reporter assay (Promega, Madison, WI, USA) was used to detect the luciferase activities.

### Pull-down assay

The pull-down assay with biotinylated RNA was performed according to the reported method [18]. Biotinylated miR-128-3p (Bio-miR-128-3p) or miR-Ctrl (Bio- miR-Ctrl) mimics were transfected into Hela and C33A cells using Lipofectamine 2000. The cells were treated with lysis buffer (Life Technologies, Carlsbad, CA, USA) and incubated with C-1 magnetic beads (Life Technologies) at 4 °C overnight. Finally, interacted RNAs were purified and detected by qRT-PCR.

### In vivo experiments

The animal experiments were approved by Experimental Animal Ethics Committee of our hospital. BALB/c nude mice (female, 5 weeks-old) were ordered from Laboratory Animal Center of Henan Province (Zhengzhou, China) and randomly separated into sh-ctrl or sh-circ-ABCB10 group. Each group contained 6 mice. C33A cells with sh-ctrl or sh-circ-ABCB10 (5 × 10^6^ / mouse) were injected into the subcutaneous tissues of mice in sh-ctrl or sh-circ-ABCB10 group, respectively. Xenograft volume was monitored every other 4 days and calculated by the formula: volume = (length × width^2^)/2. Tumor tissues were resected and weighed at day 35 after inoculation. MiR-128-3p level in CC xenograft tumors was examined through RT-qPCR assay. ZEB1, E-cadherin, N-cadherin, Vimentin and Snail expression patterns in CC xenograft tumors were measured by western blotting assay. Ki67 protein expression pattern in xenograft tumors was analyzed through immunohistochemistry (IHC) assay. IHC assay was carried out as previously described [19] with the Ki67 antibody (1/ 50 dilution, ab15580, Abcam).

### Statistical analysis

Data were analyzed by GraphPad Prism software (version 7.0, GraphPad, San Diego, CA, USA) with the outcomes expressing as mean ± standard deviation. Student’s *t*-test or one-way ANOVA along with Tukey’s post-hoc test was used to test the statistical difference between/among groups. Difference was defined as statistically significant at *P* < 0.05.

## Results

### Circ-ABCB10 expression in CC tissues and cells and its correlation with clinicopathologic parameters of CC patients

**Fig. 1:**
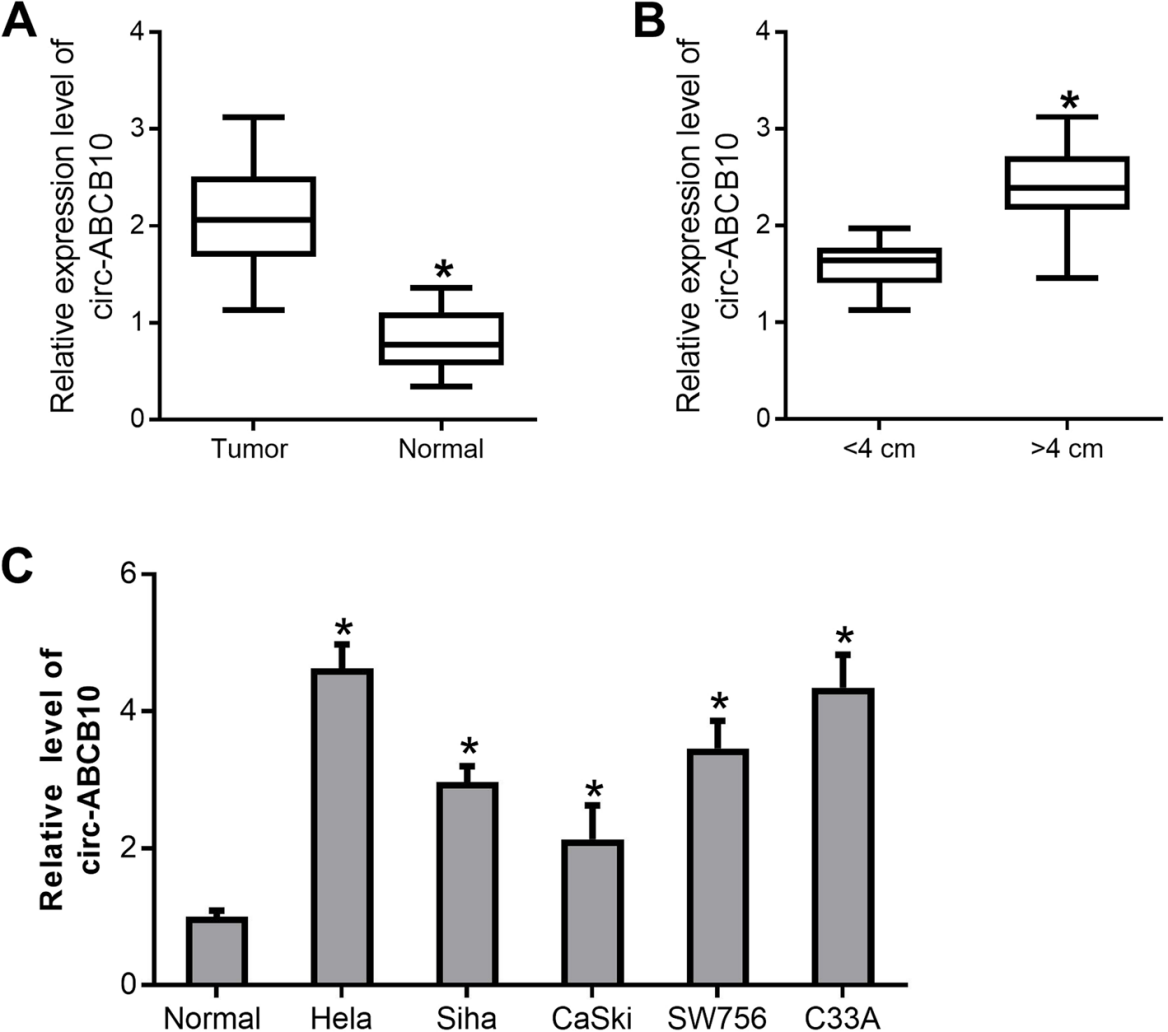
Circ-ABCB10 expression analyses in CC tissues and cells. **A-C** Circ-ABCB10 expression levels were examined by RT-qPCR assays in CC tissues and adjacent normal tissues (**A**), CC tumors with different size (**B**), 5 CC cell lines and a normal cervical cell line (**C**). *P < 0.05

Circ-ABCB10 expression was markedly up-regulated in CC tumor tissues (*n* = 34) versus adjacent normal tissues (*n* = 34) (Fig. 1A). Moreover, higher circ-ABCB10 expression was observed in CC tumors larger than 4 cm (*n* = 13) relative to tumors equal to or less than 4 cm in size (*n* = 21) (Fig. 1B). Additionally, circ-ABCB10 was highly expressed in five CC cell lines (C33A, Hela, Siha, CaSki and SW756) versus normal cervical epithelial cell line HUCEC (Fig. 1C). Furthermore, circ-ABCB10 expression was associated with tumor size, lymph node metastasis, and the International Federation of Gynecology and Obstetrics (FIGO) stage in CC (Table 1).

### Circ-ABCB10 knockdown weakened cell proliferative and invasive potential, inhibited epithelial-mesenchymal transition (EMT) and induced apoptosis in CC cells

**Fig. 2:**
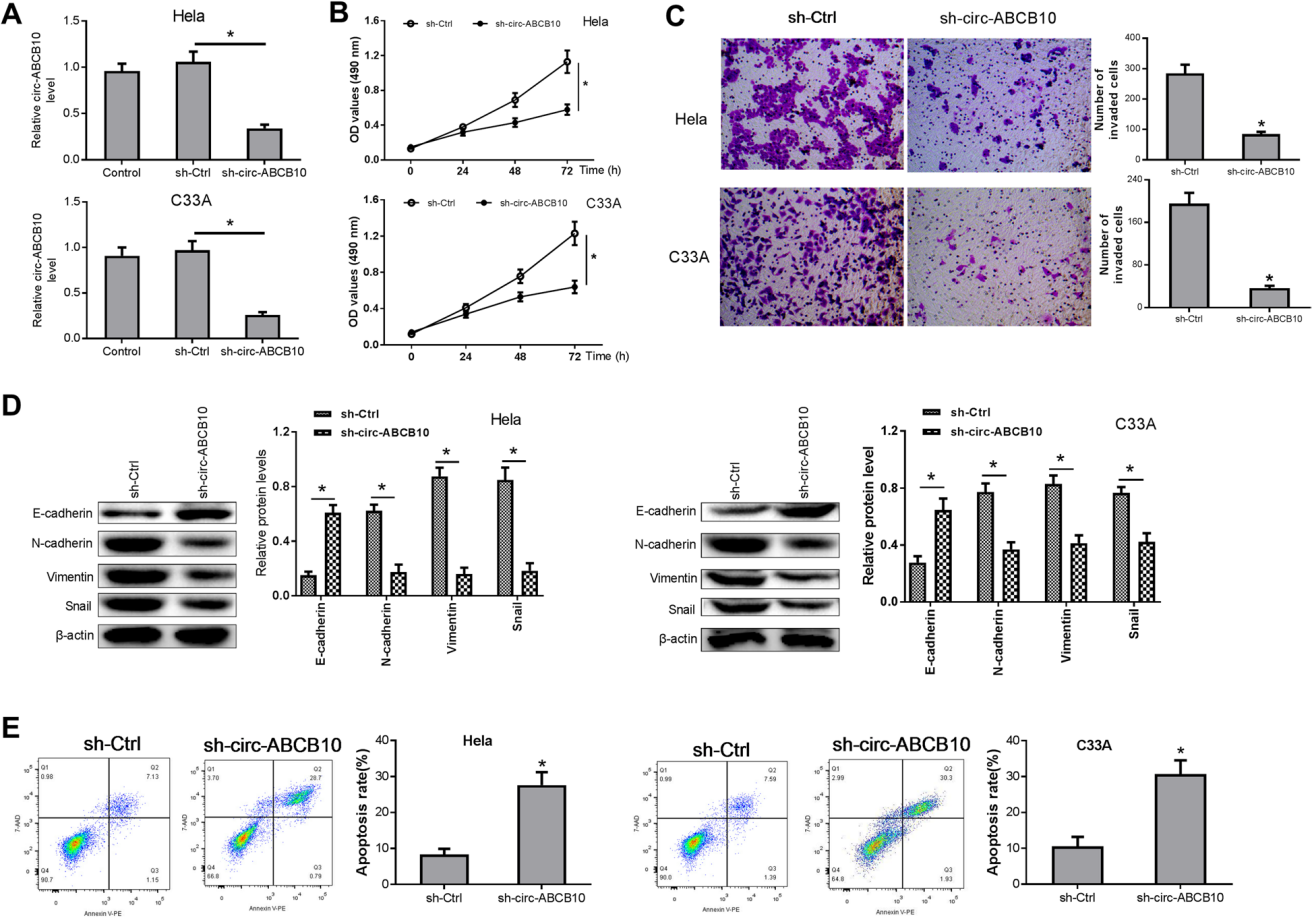
Loss of circ-ABCB10 suppressed cell proliferation, invasion and EMT and induced apoptosis in CC cells. **A-E** Hela and C33A cells were infected with sh-Ctrl or sh-circ-ABCB10 lentiviruses. **A** Circ-ABCB10 expression levels were examined through qRT-PCR method. **B** Cell proliferative activity was estimated through MTT assays. **C** Cell invasive ability was assessed through transwell invasion assays. **D** E-cadherin, N-cadherin, Vimentin, and Snail production levels were measured through western blotting assays. **E** Apoptosis was assessed using flow cytometry analyses. *P < 0.05

Moreover, circ-ABCB10 level was demonstrated to be noticeably decreased in Hela and C33A cells transduced with sh-circ-ABCB10 lentiviruses compared to sh-ctrl group (Fig. 2A). Subsequent MTT experiments showed that circ-ABCB10 depletion notably weakened the proliferative capability of Hela and C33A cells (Fig. 2B). In Fig. 2C, circ-ABCB10 knockdown markedly hampered cell invasion in Hela and C33A cells. Moreover, circ-ABCB10 silencing triggered the notable up-regulation of E-cadherin level and conspicuous reduction of N-cadherin, Vimentin, and Snail protein expression in Hela and C33A cells (Fig. 2D), suggesting that circ-ABCB10 knockdown inhibited EMT process in CC cells. Additionally, a marked elevation in cell apoptotic rate was observed in circ-ABCB10-depleted Hela and C33A cells relative to sh-ctrl group (Fig. 2E). These outcomes suggested that circ-ABCB10 depletion notably inhibited the malignant functions of CC cells.

### Circ-ABCB10 directly curbed miR-128-3p expression in CC cells

**Fig. 3:**
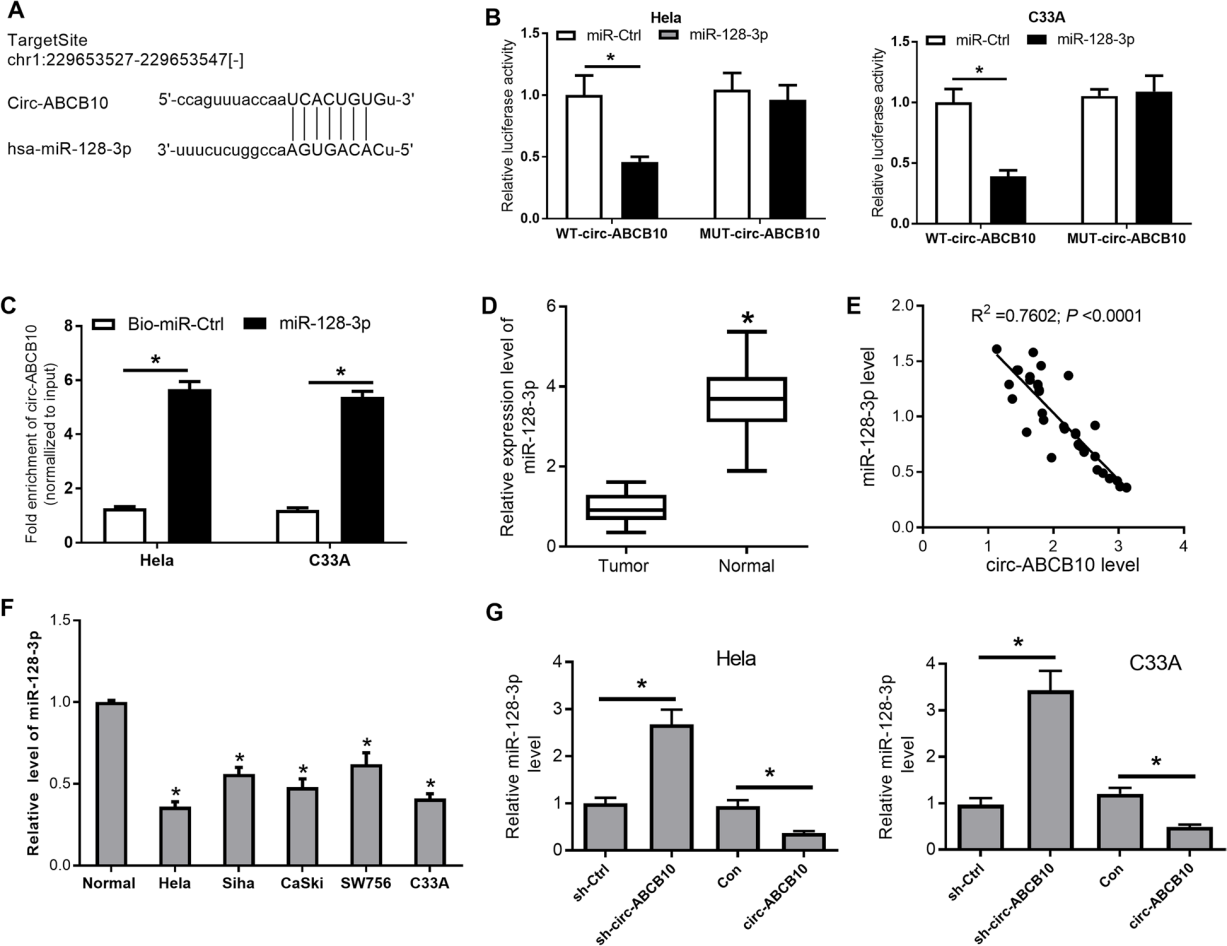
Circ-ABCB10 served as a molecular sponge of miR-128-3p in CC cells. **A** Putative complementary sites between circ-ABCB10 and miR-128-3p. **B** Luciferase activities were measured at 48 h post transfection. **C** The interaction between circ-ABCB10 and miR-128-3p in cells was further confirmed by pull-down assays. **D** The levels of miR-128-3p in CC tissues (*n* = 34) by RT-qPCR assay. **E** A negative correlation between miR-128-3p and circ-ABCB10 expressions in CC tissues. **F** MiR-128-3p level was determined by RT-qPCR assay in CC cells. **G** The effects of circ-ABCB10 loss or overexpression on miR-128-3p expression were tested through RT-qPCR assays. *P < 0.05

Next, bioinformatics prediction analyses by Starbase v2.0 software revealed that miR-128-3p had a possibility to interact with circ-ABCB10 (Fig. 3A). To further investigate the potential binding activity between miR-128-3p and circ-ABCB10, the impact of miR-128-3p up-regulation on luciferase activity of circ-ABCB10-WT or circ-ABCB10-MUT reporter was measured in Hela and C33A cells. The outcomes disclosed that miR-128-3p increase markedly reduced the luciferase activity of circ-ABCB10-WT reporter, but did not influence the luciferase activity of circ-ABCB10-MUT reporter in Hela and C33A cells (Fig. 3B). These outcomes suggested that miR-128-3p could bind with circ-ABCB10 through putative complementary sites in CC cells. Results from pull-down assays showed that circ-ABCB10 was significantly enriched in the pellet pulled down by miR-128-3p compared with control group (Fig. 3C). Moreover, miR-128-3p expression was demonstrated to be conspicuously reduced in CC tumor tissues (*n* = 34) than that in adjacent normal tissues (*n* = 34) (Fig. 3D), and to be reversely associated with circ-ABCB10 expression in CC tissues (Fig. 3E). Also, miR-128-3p was lowly expressed in CC cells (Hela, Siha, CaSki, SW756 and C33A) compared to normal cervical epithelial cells (Fig. 3F). Furthermore, circ-ABCB10 knockdown induced the marked increase of miR-128-3p expression in Hela and C33A cells (Fig. 3G). Conversely, the enforced expression of circ-ABCB10 caused the noticeable decrease of miR-128-3p level in Hela and C33A cells (Fig. 3G). In summary, these outcomes showed that circ-ABCB10 negatively regulated miR-128-3p expression by direct interaction in CC cells.

### miR-128-3p attenuated cell proliferative and invasive potential, inhibited cell EMT, and promoted cell apoptosis and circ-ABCB10 reversed the effects of miR-128-3p in CC

**Fig. 4:**
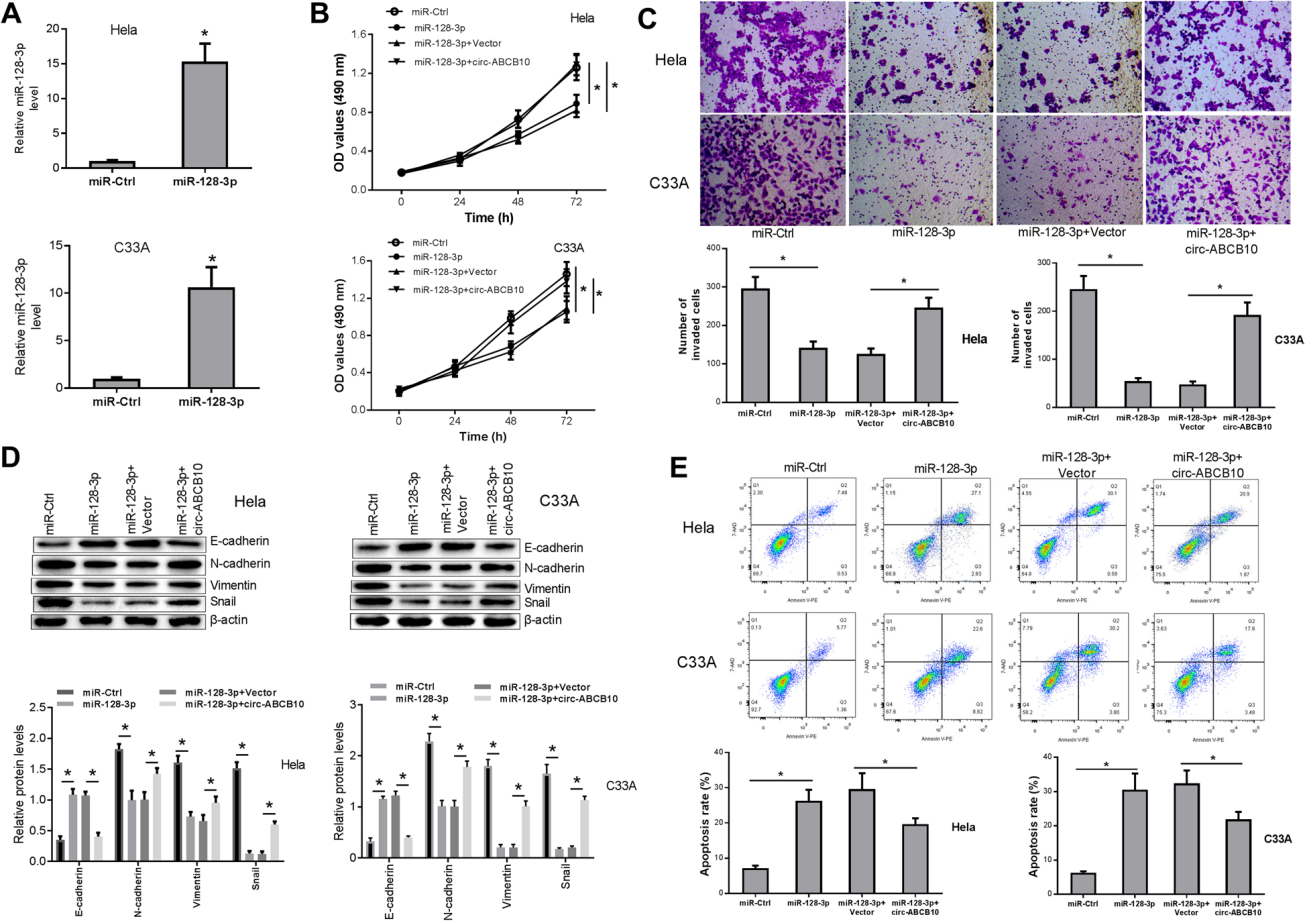
miR-128-3p curbed cell proliferation, invasion, and EMT and promoted cell apoptosis and circ-ABCB10 reversed those effects in CC. **A** MiR-128-3p levels were examined through RT-qPCR assays in Hela and C33A cells infected with miR-128-3p or miR-Ctrl lentiviruses. **B-E** Hela and C33A cells were infected with miR-128-3p or miR-Ctrl along with control or circ-ABCB10 lentiviruses, followed by the detection of cell proliferative (**B**) and invasive (**C**) abilities, EMT marker expression levels (**D**) and cell apoptotic rate (**E**). *P < 0.05

Next, RT-qPCR assays validated the marked increase of miR-128-3p level in Hela and C33A cells infected with lentiviruses expressing miR-128-3p (Fig. 4A). Functional restoration experiments showed that there was significant repression on cell proliferation (Fig. 4B), invasion (Fig. 4C) and EMT (Fig. 4D), and increase in apoptosis (Fig. 4E) by treatment of miR-128-3p in Hela and C33A cells, whereas the effects of miR-128-3p on cell functions were partially abrogated by circ-ABCB10 upregulation in Hela and C33A cells (Fig. 4B-E).

### miR-128-3p directly targeted to inhibit ZEB1 and circ-ABCB10 promoted ZEB1 expression by sponging miR-128-3p in CC cells

**Fig. 5:**
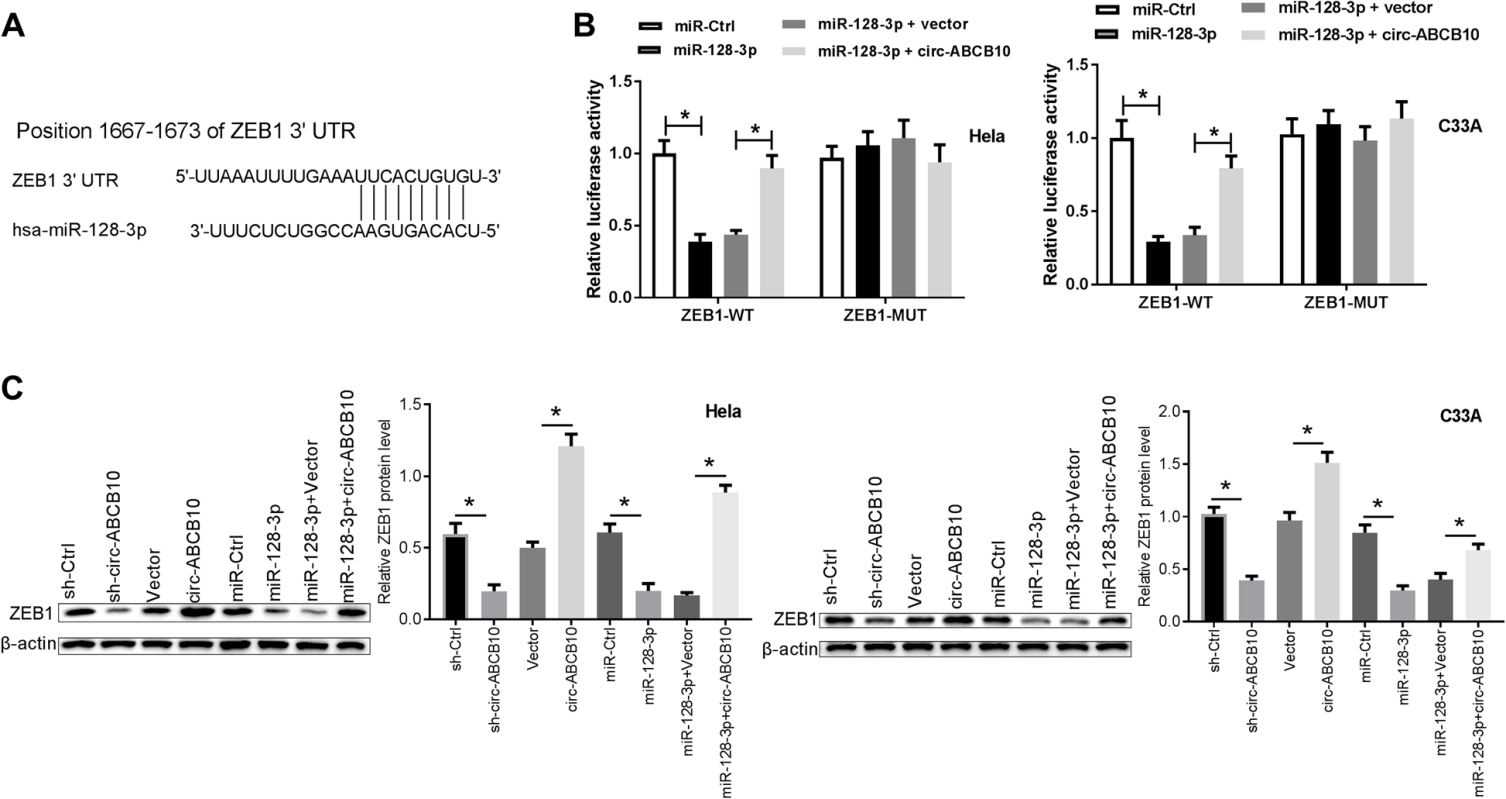
Circ-ABCB10 promoted ZEB1 expression by acting as a molecular sponge of miR-128-3p in CC cells. **A** Putative binding sites between miR-128-3p and ZEB1 3’UTR. **B** Luciferase activities were measured at 48 h after transfection. **C** ZEB1 protein levels were examined through western blotting assays at 48 h after transfection. *P < 0.05

Further, the potential targets of miR-128-3p were searched by bioinformatics prediction analyses by Starbase v2.0, TargetScan and PicTar softwares. Results suggested that there were some complementary sites between miR-128-3p and ZEB1 3’UTR (Fig. 5A). Following experiments further demonstrated that luciferase activity of ZEB1-WT reporter was noticeably reduced in Hela and C33A cells following the up-regulation of miR-128-3p (Fig. 5B). Whereas, circ-ABCB10 increase abrogated the inhibitory effect of miR-128-3p on luciferase activity of ZEB1-WT reporter in Hela and C33A cells (Fig. 5B). However, the increase of miR-128-3p alone or in combination with circ-ABCB10 did not alter the luciferase activity of ZEB1-MUT reporter with mutant miR-128-3p binding sites in Hela and C33A cells (Fig. 5B). Western blotting assays further showed that circ-ABCB10 knockdown or miR-128-3p overexpression triggered the notable reduction of ZEB1 protein level, whereas the enforced expression of circ-ABCB10 markedly raised ZEB1 protein levels in Hela and C33A cells (Fig. 5C). Additionally, circ-ABCB10 overexpression lessened the suppressive effect of miR-128-3p on ZEB1 expression in Hela and C33A cells (Fig. 5C). In summary, these data demonstrated that circ-ABCB10 facilitated ZEB1 expression by sequestering miR-128-3p from ZEB1 in CC cells.

### Circ-ABCB10 depletion impeded the tumorigenesis and EMT of CC xenograft tumors through miR-128-3p/ZEB1 axis

**Fig. 6:**
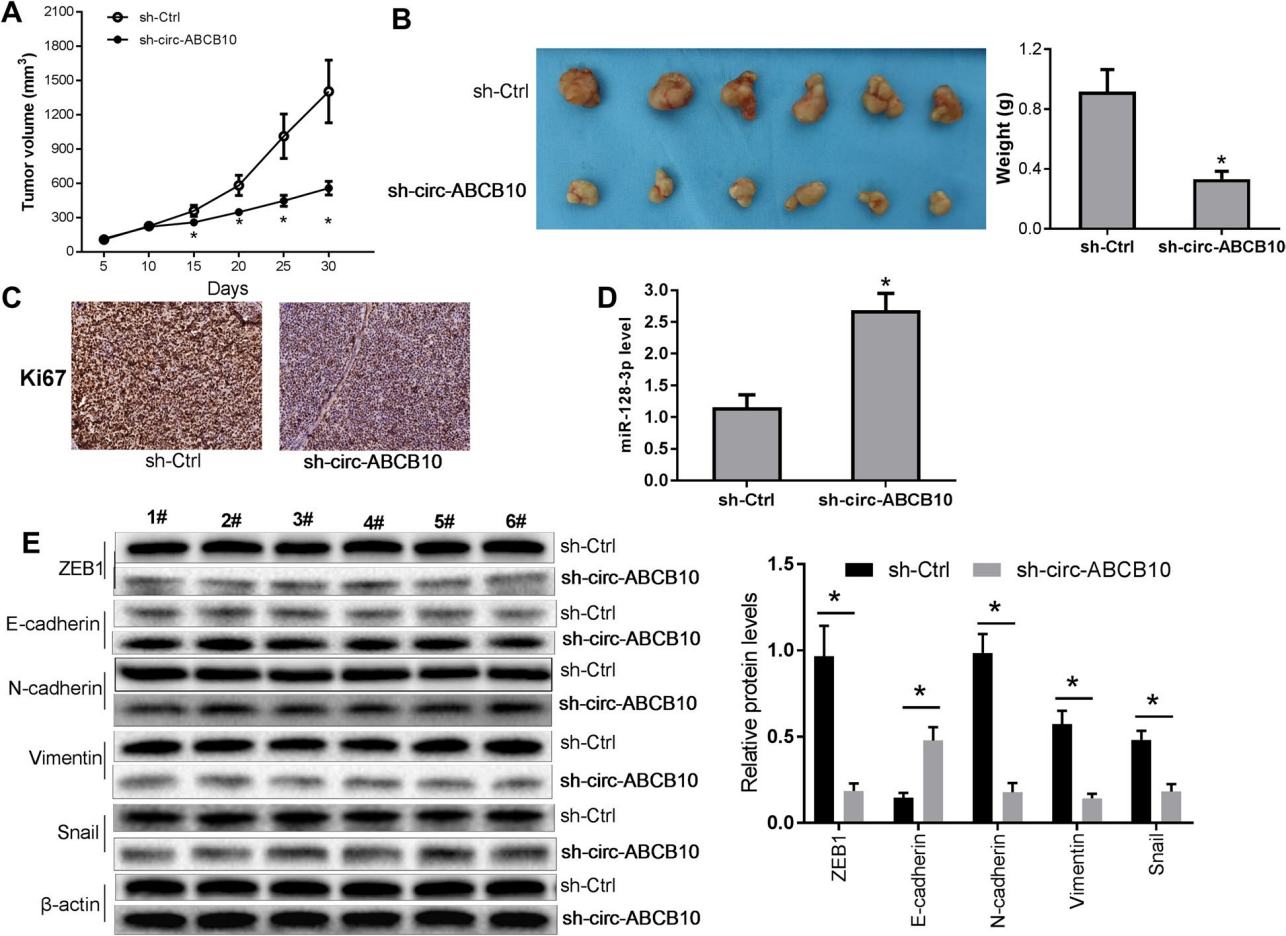
ABCB10 depletion inhibited the growth and EMT of CC xenograft tumors via miR-128-3p/ZEB1 axis*.* Mice were randomly divided into sh-ctrl or sh-circ-ABCB10 group with 6 mice per group. C33A cells stably transduced with sh-ctrl or sh-circ-ABCB10 lentiviruses were inoculated into the subcutaneous tissues of mice in sh-ctrl or sh-circ-ABCB10 group, respectively. **A** The tumor volume was monitored and recorded every 5 days. **B** At day 30, tumors were resected and weighed. **C** IHC analyses for Ki67 in xenograft tumors in sh-Ctrl and sh-circ-ABCB10 groups. **D** MiR-128-3p levels were tested through RT-qPCR assays in xenograft tumors. **E** ZEB1, E-cadherin, N-cadherin, Vimentin and Snail in xenograft tumors production levels were examined through western blotting assays. *P < 0.05

In vivo experiments further revealed that circ-ABCB10 depletion caused the notable reduction of tumor volume and weight in CC xenograft tumors (Fig. 6A and B). Moreover, Ki67 (a proliferative marker) level was noticeably reduced in CC xenograft tumors following circ-ABCB10 knockdown (Fig. 6C). Additionally, our data disclosed that circ-ABCB10 depletion led to the marked increase in miR-128-3p level and E-cadherin protein expression and conspicuous decrease in the protein levels of ZEB1, N-cadherin, Vimentin and Snail in xenograft tumors derived from C33A cells (Fig. 6D and E). These outcomes suggested that circ-ABCB10 loss hampered the growth and EMT of CC xenograft tumors via miR-128-3p/ZEB1 axis in vivo.

## Discussion

It is reported that the biological functions of circRNAs can be achieved by multiple mechanisms of action [6, 8]. One of the common molecular mechanisms is that circRNAs can positively regulate target gene expression by serving as the sponges or decoys of microRNAs (miRNAs) [6, 20].

miRNAs is a group of short non-coding RNAs that can regulate the expression of specific target genes [21, 22]. Over the past decades, miRNAs have evoked great deal of researchers’ interest by virtue of their central roles in almost all biological processes such as cell growth and death [23, 24]. Moreover, plenty of miRNAs have been documented to be implicated in cancer development by serving as oncogenic or anti-tumor factors in multitudinous malignancies including CC [25, 26]. For example, ectopic expression of microRNA-187 weakens the proliferative potential of CC cells, induces CC cell apoptosis and hinders CC xenograft tumor growth through targeting fibroblast growth factor 9 [27]. MicroRNA-155-5p promots the tumorigenesis and metastasis of CC through targeting tumor protein p53-inducible nuclear protein 1 [28]. In our study, circ-ABCB10 was determined to be upregulated in CC tissues and cells. Moreover, higher production level of circ-ABCB10 was close related to lymph-node metastasis, FIGO stage, and tumor size in CC patients. Thus, potential miRNAs that might be regulated by circ-ABCB10 were searched by bioinformatics analysis. Among these miRNAs, miR-128-3p was picked out for further investigations owing to its anti-tumor effects in some cancers such as breast cancer [29], anaplastic thyroid cancer [30] and esophageal squamous cell cancer (ESCC) [31]. For instance, miR-128-3p overexpression reduces cell proliferative, migratory and invasive potential and hampers cell cycle progression through targeting LIM domain kinase 1 in breast cancer [29]. In the subsequent experiments, our data disclosed that circ-ABCB10 curbed miR-128-3p expression by directly interaction in CC cells. Moreover, lower miR-128-3p expression was observed in CC tissues and cell lines. Furthermore, miR-128-3p upregulation weakened cell proliferative and invasive potential, hindered EMT and induced apoptosis in CC. Circ-ABCB10 up-regulation partially abrogated miR-128-3p-mediated anti-proliferation, anti-invasion, anti-EMT and pro-apoptosis effects in CC. Consistent with our outcomes, two recent studies have also shown that miR-128-3p overexpression weakens the proliferative, migratory and invasive potential of CC cells [32, 33].

Next, bioinformatics analysis was performed to search for the potential targets of miR-128-3p. Among these targets, ZEB1 was validated to be a direct target of miR-128-3p in ESCC [31]. ZEB1 has been identified as a pleiotropic transcription factor that can dominate cell fate and regulate tumor malignant progression in various cancers [34, 35]. Moreover, previous studies have revealed that ZEB1 depletion hinders the malignant progression of CC cells [36, 37]. Additionally, the aberrant expression of ZEB1 has been found to be associated with tumor differentiation, lymph node metastasis, vascular invasion and EMT in CC [38].

Thus, we further investigated whether ZEB1 was a downstream target of circ-ABCB10/miR-128-3p axis in CC. Our data presented that circ-ABCB10 facilitated ZEB1 expression by sequestering miR-128-3p from its direct target ZEB1 in CC cells. In addition, in vivo experiments demonstrated that loss of circ-ABCB10 led to the notable up-regulation of miR-128-3p level and marked down-regulation of ZEB1 expression in CC xenograft tumors. miRNAs modulate gene expression, mainly through translational inhibition or degradation of messenger RNAs (mRNA) and the biology of miRNAs is complex. Our results showed that miR-128-3p directly targeted to inhibit ZEB1. However, according to the reported studies [18], CDK14 is a target of miR-128-3p and miR-128-3p downregulation increases CDK14 expression and thus promotes cell proliferation, migration, invasion and inhibits apoptosis in ovarian cancer. According to the above results, addition of circ-ABCB10 resulted in reduction of miR-128-3p, possibly targeted different genes, and thus did not completely restore invasive/EMT/anti-apoptotic ability. Thus, some alternative reasons/pathways involved that is complex and unsure and it needs to perform further study to elucidate that.

## Conclusions

In summary, our outcomes demonstrated that circ-ABCB10 knockdown hindered the tumorigenesis and progression of CC by miR-128-3p/ZEB1 axis. To date, this is the first study to elucidate the functions and related mechanisms of action of circ-ABCB10 in CC progression. Moreover, the establishment of circ-ABCB10-miR-128-3p-ZEB1 regulatory network can deepen our understanding on CC pathogenesis, which contributes to the better management of CC. Additionally, the abnormal expression of circ-ABCB10 and miR-128-3p in CC tumor tissues and cell lines indicates the potential diagnostic value of circ-ABCB10 and miR-128-3p in CC.

## Data Availability

All data generated or analyzed during this study are included in this published article.

## Abbreviations

circRNAs: Circular RNAs
CC: Cervical cancer
EMT: Epithelial-mesenchymal transition
ZEB1: Zinc finger E-box binding homeobox 1
LMIC: Low-income and middle-income countries
ATCC: American Type Culture Collection
FBS: Fetal bovine serum
OD: Optical density
ZEB1: Zinc finger E-box binding homeobox 1
IHC: Immunohistochemistry
FIGO: Federation of Gynecology and Obstetrics
ESCC: Esophageal squamous cell cancer
