# Correction: Circ‑ABCB10 knockdown inhibits the malignant progression of cervical cancer through microRNA‑128‑3p/ZEB1 axis

**DOI:** 10.1186/s12575-023-00216-z

**Published:** 2023-08-04

**Authors:** Wei Feng, Dongya Zhang, Ruitao Zhang

**Affiliations:** Department of Gynecology, The First Affiliated Hospital of Zhengzhou University, NO.1 East Jianshe Road, Zhengzhou, 450052 Henan China

**Correction: Biol Proced Online 23, 17 (2021)**

10.1186/s12575-021-00154-8

In the original publication of this article [1], the authors identified an error the author Ruixia Guo should be removed due to lack of contribution. The original article has been corrected.
